# Synthesizing Evidence on Adverse Events of Perioperative Clonidine: An Umbrella Review: Short title: Perioperative Clonidine, Bradycardia and Hypotension

**DOI:** 10.31661/gmj.v15i.3537

**Published:** 2026-01-30

**Authors:** Mehrdad Sayadinia, Pourya Adibi, Tayyebeh Zarei, Mehrdad Malekshoar, Majid Vatankhah, Mohammad Sadegh Sanie Jahromi, Amir Hossein Pourdavood, Mansour Deylami, Lohrasb Taheri, Bibi Mona Razavi, Reza Sahraei, Shahram Shafa

**Affiliations:** ^1^ Department of Surgery, Faculty of Medicine, Hormozgan University of Medical Sciences, Bandar Abbas, Iran; ^2^ Department of Anesthesiology, Critical Care and Pain Management Research Center, Hormozgan University of Medical Sciences, Bandar Abbas, Iran; ^3^ Department of Anesthesiology, Critical Care and Pain Management Research Center, Faculty of Medicine, Hormozgan University of Medical Sciences, Bandar Abbas, Iran; ^4^ Research Center for Social Determinants of Health, Jahrom University of Medical Sciences, Jahrom, Iran; ^5^ Department of Anesthesiology, Faculty of Medicine, Golestan University of Medical Sciences, Gorgan, Iran

**Keywords:** Clonidine Adverse Effects, Perioperative Care, Bradycardia, Hypotension, Anesthesia Adverse Effects

## Abstract

**Background:**

Clonidine, a potent α2-adrenoceptor agonist, is used in anesthesia for its sedative and analgesic properties, but its administration requires careful monitoring due to potential risks of hypotension and bradycardia. As there are increasing number of trials on clonidine, this study aimed to synthesize a conclusion on unwanted consequences of clonidine use in the perioperative period.

**Materials and Methods:**

This umbrella review synthesizes evidence on adverse events associated with perioperative prescription of clonidine, following the PRIOR guidelines using three prominent databases of Scopus, PubMed, and Web of Science with a strategic combination of keywords. Studies included were systematic reviews, meta-analyses, and network meta-analyses published in English; studies reviewing prescribing systematic clonidine perioperative; and reporting adverse events. Primary outcomes were cardiovascular events, respiratory, neurological, and gastrointestinal complications. Data were extracted systematically by two independent reviewers and analyzed using random effects models in Stata. Pooled odds ratios and mean differences were calculated, with heterogeneity assessed using the I² statistic.

**Results:**

Our umbrella review of 8 systematic review studies including 223 studies from 1980 till now; all of which included only randomized controlled trials (RCTs). The bradycardia analysis, comprising four studies, shows a significant increased risk of bradycardia compared to control (OR: 1.653, 95% CI: 1.013 to 2.700) with some heterogeneity (P=0.0609). Hypotension meta-analysis (four studies) reveals a substantial increased risk (exp(theta): 3.281, 95% CI: 1.696 to 6.347), albeit with moderate heterogeneity (I^2=62.37%, P=0.0355). Heart rate reduction (two studies) and MAP reduction (two studies) meta-analyses indicate significant effects of clonidine, with substantial heterogeneity.

**Conclusion:**

Our comprehensive umbrella review highlights significant associations between perioperative clonidine and complications such as bradycardia and hypotension, suggesting the need for careful consideration and further investigation in clinical practice.

## Introduction

Clonidine as an α2-adrenoceptor agonist, has become a popular pharmacological agent holding significant implications within the context of anesthesia. While clonidine demonstrates a high affinity for α2 receptors compared to α1 receptors, its utilization extends beyond anesthesia, encompassing a spectrum of medical scenarios. Notably, clonidine is recognized for its capacity to induce sedation and allay anxiety as a premedication adjunct before surgical procedures [1]. Furthermore, when combined with local anesthetics, clonidine extends the duration of analgesia and motor blockade, thus emerging as a valuable component in postoperative pain management [2]. However, caution is warranted, especially in patients with a history of cardiovascular disease, as clonidine’s propensity to induce hypotension and bradycardia may pose risks, necessitating judicious monitoring and administration protocols [3]. While the literature underscores clonidine’s efficacy in stabilizing hemodynamics perioperatively, rigorous surveillance remains imperative to mitigate potential cardiovascular complications, including myocardial infarction [4]. Moreover, its utility extends beyond traditional applications, as evidenced by our recent study, which demonstrated that clonidine can also provide a dry surgical site during septorhinoplasty [5]. A 2016 investigation revealed that administering low-dose clonidine to patients undergoing non-cardiac surgery did not confer a survival benefit or reduce the risk of non-fatal heart attacks. However, it did increase the likelihood of developing severe hypotension and non-fatal cardiac arrests, highlighting the need for caution when using this medication in perioperative care [6]. An investigation discovered that minimal-dose clonidine heightened the likelihood of clinically significant hypotension and non-lethal heart arrest in individuals undergoing non-heart surgery [7]. Additionally, a study examining the impact of clonidine or midazolam premedication on perioperative reactions during ketamine anesthesia found that clonidine reduced pre- and postoperative blood catecholamine levels [8]. In fact, some evidences have shown that clonidine can increase systolic blood pressure in patients with severe idiopathic orthostatic hypotension [9]. On the other hand, we can see in literature that it can contribute to hypotension as a side effect, particularly in patients with certain medical conditions or when used in combination with other medications [10][11]. So, in this study we sought to amalgamate insights from available systematic evaluations and meta-analyses to illuminate the adverse outcomes of clonidine administration during the perioperative phase. Additionally, what distinguishes this investigation is its overarching review methodology, which furnishes a superior degree of evidence by aggregating data from numerous systematic assessments, thus providing a more solid and extensive grasp of the hazards linked to perioperative clonidine usage, ultimately influencing medical practice and steering subsequent research.

## Materials and Methods

This was an umbrella review study conducted based on the Preferred Reporting Items for Overviews of Reviews (PRIOR) [12]. The primary objective was to synthesize evidence on adverse events associated with perioperative clonidine use. The PICO criteria (Population, Intervention, Comparator, Outcomes) was used to determine eligibility criteria, as shown in Table-1.

### Population (P)

Inclusion: Patients undergoing surgical procedures who received perioperative clonidine.

Exclusion: Patients undergoing non-surgical procedures or those receiving clonidine for non-perioperative indications.

### Intervention (I)

Inclusion: Administration of clonidine during the perioperative period, regardless of dosage but method of administration should be systematic like oral or intravenous.

Exclusion: Studies that do not specify the timing of clonidine administration relative to the surgical procedure. No intrathecal or intraarticular injection studies were recruited.

### Comparator (C)

Inclusion: Studies comparing perioperative clonidine to placebo, no intervention, or other medications used for similar indications (e.g., other alpha-2 agonists, opioids).

### Outcomes (O)

Inclusion: Adverse events and complications associated with perioperative clonidine use.

Exclusion: Studies focusing solely on the efficacy of clonidine for pain management or other non-adverse event outcomes without detailed reporting on adverse events.

Studies with design of Systematic reviews, meta-analyses, or network meta-analyses were included. Only studies published in English with no restrictions on publication date were included.

Primary outcomes were Cardiovascular events (e.g., hypotension, bradycardia), Respiratory complications, Neurological effects (e.g., sedation, dizziness), Gastrointestinal effects (e.g., nausea, vomiting), or any other reported adverse events or side effects.

Search strategy for PubMed was "(English[lang]) AND ("systematic review" OR "systematic literature review" OR systematic OR meta-analysis OR meta-analysis OR "meta-analysis" OR meta-analyses OR "meta analyses" OR "pooled analysis" OR "pooled analyses" OR "pooled data" OR "network meta-analysis" OR "network meta-analysis") AND ("clonidine" OR "perioperative clonidine") AND (surgery OR surgical OR perioperative OR "perioperative period"). Search was conducted on May 2, 2024 till May 5, 2024.

A comprehensive literature search was undertaken, spanning multiple databases, including prominent sources such as PubMed, Scopus, Cochrane Library, Embase, Web of Science, MEDLINE, CINAHL, and Google Scholar, as well as clinical trial registries like ClinicalTrials.gov and PROSPERO. This exhaustive search aspirated to covered all available records from each database's inception up to May 5, 2024.

We used the AMSTAR (measurement tool for the 'assessment of multiple systematic reviews) criteria to evaluate the quality of the systematic reviews included in this umbrella review. This assessment ensures the reliability and validity of our findings [13].

To evaluate the overlap between the included systematic reviews, the Corrected Covered Area was estimated based on the spreadsheet prepared by Keshavarz study [14].

A uniform data extraction template was employed to systematically gather pertinent information from each of the included studies. This template was designed to capture a range of essential details, including study design elements, participant characteristics, intervention particulars, and the primary outcomes under investigation. Two independent reviewers extracted data from each study. Discrepancies between reviewers were resolved through discussion or by involving a third reviewer. Extracted data were tabulated and synthesized to provide a comprehensive overview of findings. This included qualitative synthesis for study characteristics and quantitative synthesis where applicable.

A random effects meta-analytic approach was utilized to synthesize the data, thereby accommodating both within-study and between-study variability. This enabled the derivation of more broadly applicable results. The analysis yielded pooled estimates of effect sizes, including odds ratios (ORs) for binary outcomes such as hypotension and bradycardia, as well as mean differences (MDs) for continuous outcomes, such as heart rate and mean arterial pressure, were calculated using Stata (version 17MP, StataCorp LLC). Data were extracted from eligible studies, including counts of events and means with standard deviations, and entered into Stata for analysis. The DerSimonian and Laird method was used to estimate between-study variance. Pooled ORs and 95% confidence intervals (CIs) for dichotomous outcomes were calculated using the Mantel-Haenszel method, while pooled MDs and 95% CIs for continuous outcomes were calculated using the inverse variance method. Heterogeneity was assessed using the I² statistic, with values over 50% indicating substantial heterogeneity.

## Results

**Table:** Table1. Characteristics of Included Systematic Reviews

Study ID	Study Design	Number of Included Studies	Objective	Years of Literature Review	Study Design of Included Studies	Primary Outcome	Comparison Arms	Meta-Analysis Result or Description of Comparison	Conclusion
Nishina et al. [15]	SYS REV	7	Assess efficacy of clonidine in reducing perioperative myocardial ischemic events	1980 - 1999	RCTs	Reduction of myocardial ischemia	Clonidine vs Placebo	Pooled odds ratio: 0.49 (95% CI 0.34-0.71), significant reduction in myocardial ischemia, no increase in bradycardia	Clonidine → ↓ Cardiac ischemic episodes ↔ Bradycardia
Munoz et al. [16]	SYS REV & MA	57	To evaluate the potential indications of clonidine in perioperative medicine	Not specified	RCTs	Various outcomes (analgesics consumption, nausea and vomiting, hemodynamic stability, postoperative shivering, renal and cardiac outcomes)	Clonidine vs Placebo	Pooled results: ↓ analgesics consumption, ↓ nausea and vomiting, ↓ HR, ↓ MAP, ↓ postoperative shivering	Clonidine → ↓ Pain ↔ Renal function ↔ Cardiac outcome ↓ Nausea and vomiting ↓ Shivering ↓ HR ↓ MAP
Blaudszun et al. [17]	SYS REV & MA	30	Evaluate systemic α2 agonists' effects post-surgery	1980-1999	RCT	Morphine-sparing, Pain Intensity	α2 agonists vs. Placebo	Clonidine: Weighted mean difference 4.1 mg, Dexmedetomidine: Weighted mean difference 14.5 mg	α2 agonists → ↓ Opioid consumption ↓ Pain intensity ↓ Nausea → Bradycardia Hypotension
Zhang et al. [18]	SYS REV & MA	11	Hemodynamic responses in patients undergoing laparoscopic gallbladder removal are affected by clonidine administration	Up to April 2017	RCTs	MAP and HR	Clonidine vs Placebo	Reduction in MAP, HR, propofol requirement, and PONV	↓ MAP, ↓ HR, ↓ propofol requirement, ↓ PONV
Demiri et al. [19]	SYS REV & MA	56	Adverse events in non-cardiovascular surgery under general anesthesia	2012 - 2018	RCTs	Incidence of severe adverse events	a2-adrenoceptor agonists vs Placebo	Moderate-quality evidence shows hypotension and bradycardia are common adverse events, while dexmedetomidine seems to protect against intraoperative hypertension and tachycardia	α2-adrenoceptor agonists → ↑ Hypotension ↑ Bradycardia ↓ Hypertension ↓ Tachycardia
Lee et al. [20]	SYS REV & MA	37	nasal surgery adverse events	Up to February 2020	RCTs	Intraoperative morbidity, operative time, bleeding, hypotension, PONV, postoperative pain	Hypotensive agent vs Placebo/Other agents	Dexmedetomidine shows greatest differences in intraoperative bleeding, intraoperative opioid administration, and postoperative pain compared with placebo	Dexmedetomidine → ↔ Other hypotensive agents ↑ Perioperative benefits ↓ Operative time ↓ Intraoperative bleeding ↓ Postoperative pain
Wang et al. [21]	SYS REV	14	To compare clonidine with tramadol for shivering control following spinal anesthesia.	Up to August 2020	RCTs	Shivering control effectiveness	Clonidine vs. Tramadol	Clonidine less effective (OR: 0.59; 95% CI: 0.40-0.88; p=0.009) than tramadol	Clonidine< Tramadol
Ju et al. [22]	SYS REV	11	alpha-2 agonists effect on pain and opioid consumption.	Up to March 2019	RCTs	Cumulative opioid consumption, Postoperative pain intensity	Alpha-2 agonist vs. Control (Placebo or No treatment)	Ut significantly reduces opioid consumption and pain.	↑ Heterogeneity limits certainty

Reduction ↓, Increase/Improve ↑, No Change or difference ↔, Mean Arterial Pressure MAP, Heart Rate HR, Postoperative Nausea and Vomiting PONV, Randomized Controlled Trial RCT, Systematic Review and Meta-Analysis SYS REV & MA, Odds Ratio OR,

Our umbrella review of 8 systematic review studies [15][16][17][18][19][20][21][22] including 223 individual studies from 1980 till now; all of which included only randomized controlled trials (RCTs). The calculated CCA was 0.82% that was far below the concerned level, so there was no significant overlap between the systematic review studies.

The included studies in this review (Table-1) cover a diverse range of objectives related to the use of clonidine and other α2-adrenoceptor agonists in perioperative medicine. Nishina et al. [15] focus on assessing clonidine's efficacy in reducing perioperative myocardial ischemic events, while Munoz et al. [16] aim to evaluate potential indications of clonidine in the perioperative setting. Blaudszun et al. [17] examine the effects of systemic α2 agonists post-surgery, and examined the impact of clonidine on hemodynamic stability in individuals undergoing laparoscopic removal of the gallbladder, shedding light on its effects in this specific surgical context. Demiri et al. [19] explore adverse events associated with α2-adrenoceptor agonists in non-cardiovascular surgery under general anesthesia, while Lee et al. study the adverse effects of hypotensive agents in patients undergoing nasal surgery. Research by Wang et al. [21] juxtaposed the efficacy of clonidine and tramadol in preventing shivering episodes after spinal anesthesia, while Ju et al. [22] explored the potential of preoperative alpha-2 agonist administration to mitigate postoperative pain and reduce opioid requirements. These studies collectively provide insights into the potential benefits, adverse effects, and broader applications of α2-adrenoceptor agonists in the perioperative context, contributing valuable knowledge to clinical practice and future research endeavors.

**Figure-1:**
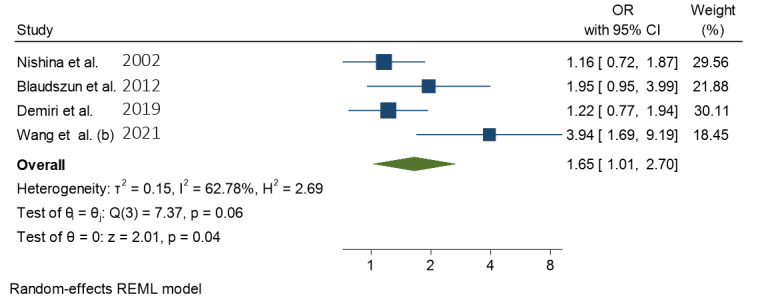
Figure 1. Forest plot of intraoperative bradycardia after clonidine administration

Nishina et al. [15] demonstrate a significant reduction in myocardial ischemia without an increase in bradycardia with clonidine administration. Munoz et al. [16] report notable benefits such as reduced analgesic consumption, nausea, vomiting, and improved hemodynamic stability, although no impact on renal or cardiac outcomes is observed. Blaudszun et al. [17] highlight differences in the effects of clonidine and dexmedetomidine on outcomes such as medication dosage. Zhang et al. [18] show significant reductions in mean arterial pressure (MAP) and heart rate (HR) with clonidine during specific perioperative phases. Demiri et al. [19] underscore common adverse events like hypotension and bradycardia, while suggesting dexmedetomidine's potential in mitigating intraoperative hypertension and tachycardia. Lee et al, [20] identify dexmedetomidine's impact on intraoperative bleeding, fentanyl administration, and postoperative pain. Wang et al. [21] contrast clonidine's efficacy with tramadol for shivering control, while Ju et al. [22] demonstrate the benefits of preoperative α2 agonist administration in reducing opioid consumption and pain intensity. These findings collectively highlight the multifaceted effects and potential clinical implications of α2-adrenoceptor agonists in perioperative care, offering insights for optimizing patient outcomes and guiding future research endeavors. Forest plot in Figure-1 presents the results of a meta-analysis assessing the effects of intraoperative clonidine versus placebo on bradycardia during surgery. With four studies included, the analysis reveals an overall effect size (OR) of 1.653 (95% CI: 1.013 to 2.700), indicating a significant impact of clonidine on bradycardia compared to placebo (P=0.0444). Despite some observed heterogeneity among the studies, the test of homogeneity does not reach statistical significance (P=0.0609). Notably, individual study effects range from 1.160 to 3.940, with corresponding confidence intervals indicating variability in the estimated effects. These findings collectively suggest that intraoperative clonidine may indeed influence bradycardia occurrence, warranting attention in clinical practice and further investigation. This forest plot summarizes a meta-analysis investigating the impact of intraoperative clonidine versus placebo on hypotension across four studies. Utilizing a random-effects model, the analysis reveals an overall effect size (OR) of 3.281 (95% CI: 1.696 to 6.347), signifying a substantial association between clonidine administration and hypotension during surgery (P=0.0004). Despite moderate heterogeneity among the studies (tau^2=0.2614, I^2=62.37%), the test of homogeneity suggests significant differences across studies (P=0.0355). Individual study effects range from 1.780 to 5.970, with corresponding confidence intervals indicating variability in the estimated effects. Notably, studies by Blaudszun et al . [17] and Wang et al. [21] contribute significantly to the overall effect size. These findings underscore the importance of careful monitoring and management of hypotension when utilizing clonidine intraoperatively, warranting further investigation and clinical consideration. This meta-analysis examines the impact of intraoperative clonidine versus placebo on heart rate reduction across two studies, employing a random-effects model. The analysis reveals an overall effect size (exp(theta)) of 8.165 (95% CI: 2.095 to 31.815), indicating a significant association between clonidine administration and heart rate reduction during surgery (P=0.0025). Notably, there is substantial heterogeneity among the studies (tau^2=0.8182, I^2=83.27%), suggesting differences in effect sizes between studies (P=0.0145). The study by Munoz et al. [16] contributes more significantly to the overall effect size compared to Zhang et al. [18] These findings emphasize the potential for marked heart rate reduction with intraoperative clonidine use, necessitating careful monitoring and consideration of individual patient factors. Further research is warranted to elucidate the clinical implications and optimize patient management strategies. This meta-analysis examines the effects of intraoperative clonidine versus placebo on mean arterial pressure (MAP) reduction across two studies, employing a random-effects model. The analysis reveals an overall effect size (exp(theta)) of 6.149 (95% CI: 1.320 to 28.639), indicating a significant association between clonidine administration and MAP reduction during surgery (P=0.0207). There is considerable heterogeneity among the studies (tau^2=1.0647, I^2=85.52%), suggesting substantial variability in effect sizes between studies (P=0.0086). Notably, the study by Munoz et al. [16] contributes more significantly to the overall effect size compared to Zhang et al. [18] These findings underscore the potential for marked MAP reduction with intraoperative clonidine use, emphasizing the importance of vigilant monitoring and tailored patient management strategies. Further research is necessary to better understand the clinical implications and optimize perioperative care practices.

**Figure-2:**
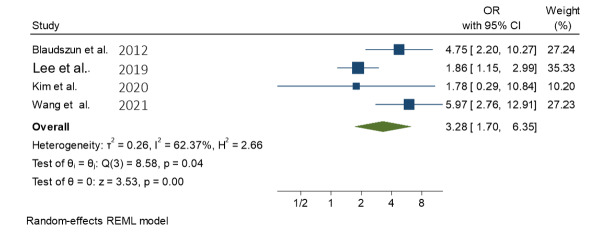
Figure 2. Forest plot of intraoperative hypotension after clonidine administration

**Figure-3:**
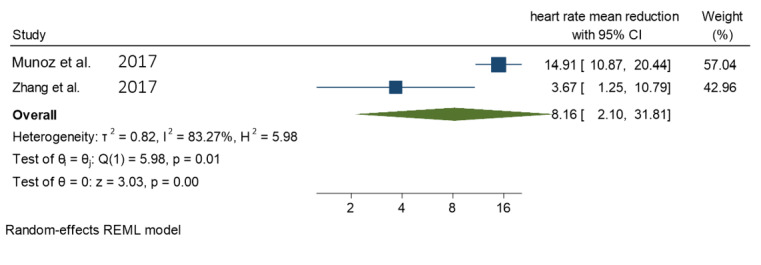
Figure 3. Forest plot of magnitude of heart rate reduction after clonidine administration

**Figure-4:**
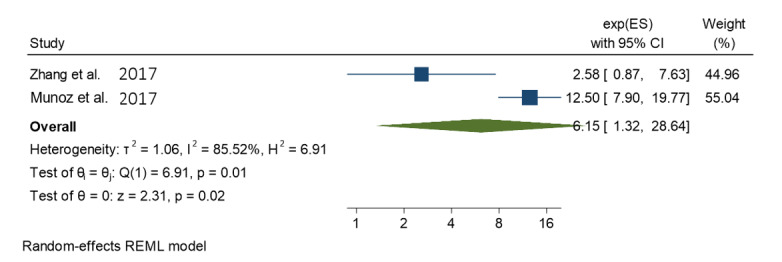
Figure 4. Forest plot of magnitude of mean arterial pressure reduction after clonidine administration

## Discussion

Our study showed a significant effect of clonidine on the incidence of intraoperative hypotension and bradycardia; while its confrere medication in the α2-adrenoceptor agonist group, dexmedetomidine, has shown protective effects against intraoperative hypertension and tachycardia [23][24][25]. This dichotomy in the effects of clonidine and dexmedetomidine underscores the nuanced role of α2-adrenoceptor agonists in perioperative management. Clonidine's propensity to cause hypotension and bradycardia reflects its potent central and peripheral sympatholytic actions, which can be advantageous in certain surgical settings but may also pose risks in patients with compromised cardiovascular stability. This was the reason that we excluded studies that included effects of α2-adrenoceptor agonists for hypotension and bradycardia together, like Wijeysundera et al.'s study [23]. However, these medications’ hypotensive effects can be counteracted by antagonists like idazoxan and efaroxan, which act on both imidazoline receptors and α2 receptors [23]. The ability to modulate these effects with antagonists shows the importance of personalized medicine in perioperative care, where the balance between therapeutic benefits and potential adverse effects must be carefully managed.

Research suggests that clonidine may reduce the occurrence of perioperative myocardial ischemia during noncardiac surgery, potentially offering benefits in certain medical contexts [24]. This finding is particularly significant for high-risk patients, where the reduction of myocardial ischemia can significantly improve surgical outcomes and reduce postoperative complications. However, there are reports indicating that abrupt withdrawal from clonidine therapy can rarely lead to acute myocardial infarction [25]. This shows the importance of gradual tapering and careful monitoring when discontinuing clonidine, especially in patients with known cardiovascular disease. Moreover, recent studies have shown that clonidine does not mitigate the risk of myocardial infarction in specific situations, such as during perioperative care [26]. This suggests that while clonidine may have protective effects in some contexts, its efficacy is context-dependent and may not be universally beneficial. Additionally, clonidine has not demonstrated effectiveness in reducing mortality or non-fatal heart attacks in the short term after randomization [27]. These findings highlight the need for a more comprehensive understanding of the mechanisms through which clonidine exerts its effects and the specific patient populations that may benefit most from its use. On the other hand, our study found that it might increase the risk of cardiac adverse events when considering results from a pool of more than 200 single studies. This comprehensive analysis suggests that the overall risk-benefit profile of clonidine in perioperative care may be more complex than previously thought, necessitating a cautious and individualized approach to its use.

An individual study on the effects of clonidine on patients with autonomic dysfunction found that the maximum decrease in systolic BP induced by clonidine was 26±6 mm Hg. This was modulated through ganglionic blockage [28]. This significant reduction in systolic blood pressure highlights the potent sympatholytic effects of clonidine, which can be particularly pronounced in patients with autonomic dysfunction. This can make the pressor response to norepinephrine and its analogues very unpredictable [29]. The unpredictability of the pressor response in these patients underscores the complexity of managing autonomic dysfunction and the need for tailored therapeutic strategies to ensure patient safety and optimal outcomes.

As well as what we concluded in our study, Hanna et al. study examined the effectiveness and safety of clonidine in rapidly lowering blood pressure in hospitalized patients with severe hypertension but no symptoms. They reviewed the medical records of 200 patients who received clonidine within 6 hours of developing severe hypertension. The goal was to see how many patients experienced a significant drop in blood pressure (at least 30% reduction in mean arterial pressure) within 4 hours of taking clonidine. The results showed that about 10% of patients achieved this significant drop in blood pressure, with women and those receiving a higher dose of clonidine (0.3 mg) being more likely to respond well. The researchers also found that older age and preexisting vascular disease were associated with a better response to clonidine. However, they noted that it was difficult to predict which patients would respond well to the medication based on their clinical characteristics. In terms of safety, the researchers observed 14 adverse events within 24 hours of clonidine administration, with most being cases of acute kidney injury [31]. The optimal dosage of clonidine for perioperative use remains a topic of investigation, with studies suggesting that a small oral dose of 0.1-0.2 mg may be effective in reducing the incidence of perioperative myocardial ischemic episodes [32][33].

## Conclusion

In conclusion, our umbrella review provides a comprehensive synthesis of evidence regarding adverse events associated with perioperative clonidine use. Through rigorous analysis of eight systematic review studies comprising 223 studies spanning over four decades, we found compelling evidence linking clonidine to adverse cardiovascular outcomes, particularly bradycardia and hypotension. These findings underscore the importance of cautious utilization of clonidine in perioperative settings and advocate for further exploration through robust clinical investigations. Clinicians should exercise prudence in prescribing clonidine, taking into account the potential risks highlighted in this review, to optimize patient safety and outcomes.

## Conflict of Interest

None.
